# 

**Published:** 1887-12-03

**Authors:** 


					PRICE ONE PENNY.
No. 62, Vol. Ill-
December 3rd, 1887.
B^gistet'ed at the Gtmeral Post^O.'Hce
as a Newspaper.]
Per Annum, 6s. 6d., post free. ^
Monthly Parts, 6d.; post free, 7id.
Ditto per Ann., 7s. 6d. post free.
Science, flfcebtdne, anb flbbtlantbcopE,
WITH EXTRA SPECIAL SUPPLEMENT.
LONDON'S PROBLEM SOLVED : Work for the Unemployed?Hope for all.

				

